# Laser-induced convection shifts size distributions in nanoparticle tracking analysis

**DOI:** 10.1039/d1na00572c

**Published:** 2021-09-01

**Authors:** William H. Hoffmann, Niall M. C. Mulkerns, Simon R. Hall, Henkjan Gersen

**Affiliations:** H. H. Wills Physics Laboratory, University of Bristol Tyndall Avenue Bristol BS8 1TL UK h.gersen@bristol.ac.uk; Bristol Centre for Functional Nanomaterials, University of Bristol Tyndall Avenue Bristol BS8 1TL UK; School of Chemistry, University of Bristol Cantock's Close Bristol BS8 1TS UK

## Abstract

This work discusses the effects of increasing laser power on the size data derived from NTA for particles of known size and scatterers in solutions of flufenamic acid in ethanol. We find that whilst a higher laser power reveals more particles as expected, their residence time changes due to laser-induced convection. This reduced residence time decreases the number of tracks available for individual particle size determination, shifting the size distribution to smaller values. This problem is overcome by using a shutter to inhibit the development of convection currents, increasing particle residence time and reducing the error on the size distribution. The detailed understanding of laser-induced convection permits more robust size characterisation of mesoscopic organic clusters, which play a key role in two-step nucleation theory.

## Abstract 2

In nanoparticle tracking analysis, laser-induced convection increases the error on individual particle sizing.

## Graphical abstract

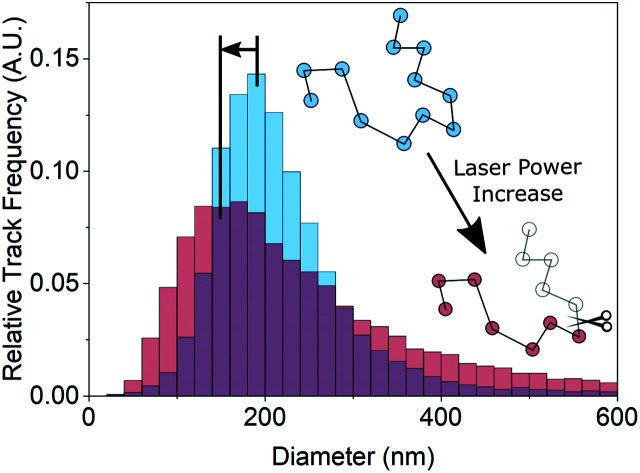

## 1. Introduction

Nanoparticle Tracking Analysis (NTA) is a technique utilised in the size characterisation of myriad nanoparticles, including extracellular vesicles,^1^ proteinaceous particles,^2^ and macromolecular assemblies.^3^ This technique analyses the Brownian motion of individual particles to generate a size distribution of the sample and can size particles in the range of 10 nm to 1000 nm.^4^ Dynamic light scattering, a related technique, outputs similar data on particle size distribution, but NTA offers key advantages including single particle characterisation, superior size peak resolution, and insensitivity to contaminants.^5^ In NTA, particles can be analysed in their liquid media without fixation protocols as typically required for other particle sizing techniques. This ability to analyse in the native particle dispersion makes NTA useful for studying mesoscopic clusters in organic crystal solutions, where the properties of the solution, *e.g.* solute concentration and temperature, have been shown to affect the mesoscopic cluster size and concentration.^6–8^ Such systems are studied because of their involvement in two-step nucleation theory, a nucleation mechanism which proceeds *via* cluster intermediates.^9^

Mesoscopic clusters present a challenge in NTA because their small size and low refractive index contrast lower the scattering irradiance. The expected Rayleigh scattering signal can be written as follows:^10^1
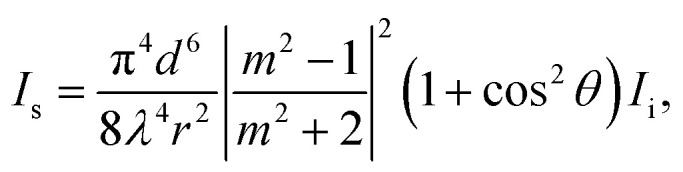
where *I*_s_ is the scattering irradiance, *d* is the particle diameter, *λ* is the wavelength, *r* is the distance from the scatterer to the detector, *m* is the refractive index contrast, *θ* is the scattering angle, and *I*_i_ is the irradiance of unpolarised incident light. A small cluster size, such as 70 nm in olanzapine-ethanol/water solutions^7^ dramatically decreases the scattering signal obtained. Additionally, refractive index contrast is reduced by solvent content within these clusters.^6,8,11,12^ The low scattering irradiance prevents the detection of small clusters in NTA, modifying the obtained size distribution due to missing particles below the detection limit. To access smaller particle populations, the incident laser power can be increased to induce a proportional increase in scattering signal from small particles as shown in eqn (1).

In this paper, we show the effects of increasing laser power on the NTA characterisation of scatterers in solutions of flufenamic acid (FFA) in ethanol. Whilst the size distribution shifts to lower values with an increase in laser power as expected, the average particle residence time, *i.e.* time spent in the field of view, decreases. We interrogate particles of known size with NTA to show that the used higher laser power induces a convection current which causes the particles to move through the field of view more quickly. More importantly, the size distribution determined by NTA shifts to smaller sizes as a result of the reduction in residence time, obscuring any improvement in the detection limit at higher laser power. Residence time is restored to larger values by incorporating a shutter in the illumination arm which inhibits the development of convection currents, resulting in improved size distributions. The detailed understanding of the impact of laser-induced convection on NTA data developed in this work permits more robust size characterisation of particles at high laser powers.

## 2. Experimental apparatus and procedure

**Fig. 1:**
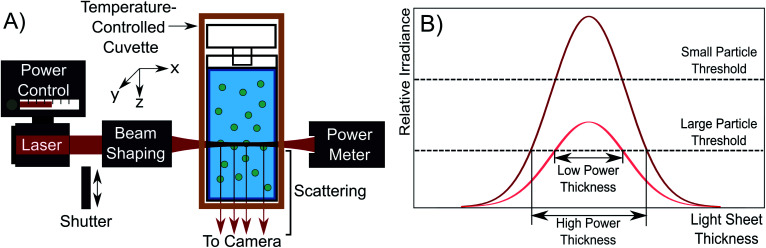
(A) A schematic of the NTA microscope used in which the laser output is power-controlled and shaped to form a light sheet. The light sheet irradiates the particle dispersion held within a temperature-controlled cuvette to induce scattering. The scattering orthogonal to the light sheet is recorded as a video. (B) A plot showing the effect of increased laser power on the light sheet. Increasing the laser power from low (pink) to high (red) power improves the detection limit and increases the volume of the solution interrogated.

The NTA microscope used in this study is a custom-built system, with control over laser power and the shape of the beam cross section as shown schematically in Fig. 1A. In this system, a 200 mW, 638 nm laser (Thorlabs LD638P200) is coupled into a single mode fibre (Thorlabs S405-XP), and light exiting the fibre is collimated with a collimator (Thorlabs CFC-8X-A). The light is sent through beam-shaping optics which flattens the cross section of the beam to a light sheet.^13^ The dimensions of the light sheet were determined with fluorescent red dye (CellTracker deep red dye) diluted with anhydrous ethanol (Sigma-Aldrich) and are approximately 220 μm × 170 μm × 30 μm along the *x*-, *y*-, and *z*-axes directions, respectively. The light sheet propagates though the nanoparticle dispersion held within a cuvette, inducing light scattering from illuminated particles. The cuvette is held within an insulated temperature-controlled copper block to hold the cuvette at (20.00 ± 0.03) °C. A power meter (Newport 818-SL/DB photodetector) was used to calibrate the laser power with the cuvette removed.

The apparatus is different from typical commercial NTA devices by having control over the laser power. The effect of increasing laser power is to increase the irradiance of the incident light sheet as shown in Fig. 1B. As laser power is increased, the light sheet irradiance increases across the whole distribution. This has the effect of both enhancing the detection limit of the device, enabling the detection of smaller particles and increasing the light sheet volume, allowing the detection of particles in a larger solution volume. The detection limit improves because the higher power density at the peak of the light sheet allows for particles without sufficient scattering irradiance in the low power case to scatter more light (eqn (1)). This is shown in Fig. 1B by the relative irradiance reaching the small particle threshold (red distribution). The threshold for detection is set by the camera exposure, which is limited by the frame rate needed to collect video which is typically 25 or 30 frames per second (FPS) for NTA.^14,15^ The usable volume of the light sheet also increases because a larger thickness of the light sheet has sufficient irradiance to induce the required scattering signal for detection of larger particles. This is depicted by the large particle threshold and effective thicknesses in Fig. 1B. Essentially, the probing depth along the *z*-axis (see Fig. 1A) increases. Because of the detection limit improvement and the probing depth increase, a higher laser power causes a greater number of particles to be visible in the field of view. It is important to note that we assume a consistent particle structure in the distribution, eliminating the need to consider particle differences in refractive index contrast across the size distribution.

Scattering is collected orthogonally to the incident laser propagation using a two lens microscope and is recorded in a video with a CMOS camera (Ximea MQ013MG-E2) at 25 FPS for FFA solutions or 30 FPS for particles of known size. The pixel size was calibrated by performing NTA on 1000 nm silica microspheres (Thermo Fisher Scientific, 8100 Duke Standards) dispersed in lab ethanol (Sigma-Aldrich) and was found to be 170 nm per pixel. A region of interest is selected in the brightest portion of the light sheet to produce a image size of approximately 220 μm × 90 μm. The collected video shows scattering centres moving with Brownian motion. Tracking analysis is performed on these scattering centres to derive size and residence time distributions using the Trackpy Python package (version 0.4.2), which is based on the Crocker–Grier tracking algorithm.^16^ Mean squared displacements for individual particles are related to the diffusion coefficient by:^4^

2 〈Δ*r*^2^〉 = 4*Dt*,

where 〈Δ*r*^2^〉 is the 2-dimensional mean squared displacement, *D* is the diffusion coefficient, and *t* is the lag time between particle displacements. A linear regression is performed on a mean-squared displacement *vs.* time plot to derive the particle diffusion coefficient using the first 2 lag times for FFA samples or 5 lag times for the particles of known size as done previously.^7^ The minimum track length is set to 2 for FFA due to its low residence time and 5 for the other particles. The size of each particle is calculated using the Stokes–Einstein relation,3
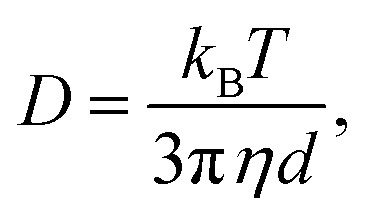
where *k*_B_ is the Boltzmann constant, *T* is temperature, *η* is solution dynamic viscosity and *d* is particle diameter. Each particle size is plotted on a size histogram scaled by the number of frames the particle is present in the field of view to prevent bias towards smaller particles. Smaller particles enter and exit the field of view more often because of their larger diffusion coefficients, meaning that more individual small particles are tracked. Scaling by particle track length eliminates this bias.^17^ Concentration is calculated by averaging the number of particles detected in each frame and dividing by the light sheet volume. Residence time is determined by multiplying the time between each frame by the number of frames the particle is present in the field of view less one. Tracking analysis parameters (*e.g.* particle signal threshold) were kept the same for all video analysis of each sample type. All errors on tracking data reported in this work are one standard error on the mean.

Particle samples were prepared as follows. Flufenamic acid (Sigma-Aldrich, analytical standard for drug analysis) was dissolved at room temperature in anhydrous ethanol at concentrations of 0.5 M and 1 M and filtered with a 0.2 μm pore size polytetrafluoroethylene filter (Fisherbrand) into a cuvette (10 mm path length, 3.5 mL capacity). The dynamic viscosity of the FFA solutions was characterised with 1000 nm Si particles dispersed in the FFA solutions as done previously;^7^ the change in viscosity is due to dissolved FFA in the solution. 40 nm gold (BBI solutions), 200 nm polystyrene (Sigma-Aldrich), and 1000 nm silica particles were all dispersed in lab-grade ethanol. Samples were placed in the cuvette holder and allowed to equilibrate prior to data collection for at least 30 min. FFA was characterised after 30 min equilibration time to control for possible population changes over time.^7,8^ A blank of ethanol was collected with the same cuvette prior to particle preparation to ensure the identity of the particles detected with the device.

## 3. Flufenamic acid in ethanol

### 3.1 0.5 M concentration

**Fig. 2:**
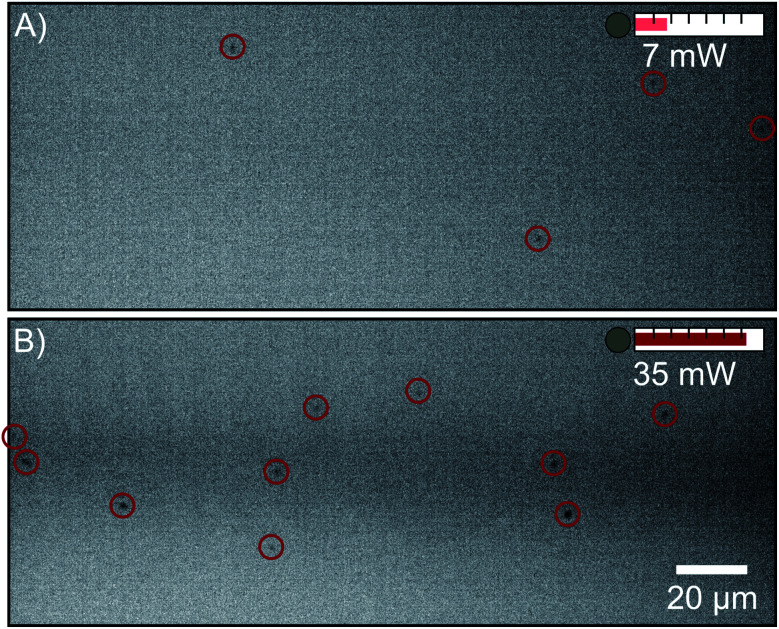
Negative video stills of 0.5 M flufenamic acid in ethanol with laser powers of (A) 7 mW and (B) 35 mW, demonstrating the increased sensitivity as a function of laser power. Particles are marked with red circles. The scatterer concentrations detected are (1.26 ± 0.07) × 10^6^ particles per mL and (5.7 ± 0.3) × 10^6^ particles per mL, respectively. The camera collection parameters are the same in each image.

Clustering in solutions of FFA and ethanol has been shown previously using liquid phase electron microscopy.^18^ NTA was performed on a 0.5 M solution of FFA and ethanol to determine if scatterers can be detected with this technique. Fig. 2 shows video stills of FFA scatterers at two different laser powers. At 7 mW, a scatterer concentration of (1.26 ± 0.07) × 10^6^ particles per mL is detected, but when the laser power is increased to 35 mW, a larger concentration of (5.7 ± 0.3) × 10^6^ particles per mL is revealed. Note that the camera collection and tracking parameters are the same and only laser power is changed between the collection of these videos. This shows that a substantial portion of the FFA scatterer population has a size below the detection limit at 7 mW. This limits the use of NTA as a tool to screen solutions of unknown mesoscopic character for scattering when using NTA systems without a sufficient laser power. Whilst there is a clear increase in particle concentration with higher laser power as would be expected in a particle dispersion, it is unclear whether this increase is caused by the observation of smaller particles due to the enhancement of the detection limit or by the observation of the same particle size range in a larger portion of the light sheet (Fig. 1B).

### 3.2 1 M concentration

**Fig. 3:**
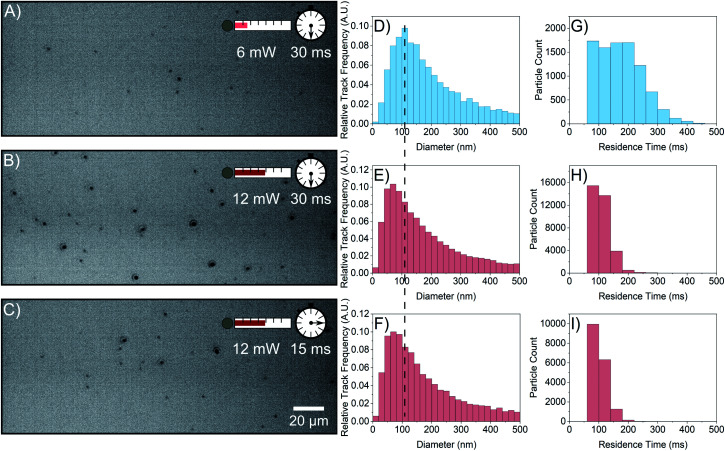
NTA of a 1 M flufenamic acid in ethanol at 6 mW and 12 mW. (A–C) Negative video stills with the labelled laser intensities and camera exposure times. (D–F) Corresponding size distributions. At 12 mW, there is a shift in the distribution toward smaller sizes. The dashed line is centred on the modal bin of the top histogram as a guide to the eye. (G–I) Corresponding residence time distributions. At 12 mW, there is a shift to the left and narrowing of the residence time distributions. The third row has a lower camera exposure time to collect scattering signal comparable to the first row to show that the change in the size and residence time distributions depend on laser power but not on scattering signal.

To understand the reason for the observed increased particle concentration, a 1 M FFA solution was interrogated with NTA. This sample displays substantial scatterer populations at both 6 mW and 12 mW laser powers as shown in Fig. 3A–C. Like the 0.5 M sample, increasing the laser power increases the concentration of scatterers detected from (1.92 ± 0.04) × 10^7^ particles per mL (Fig. 3A) to (4.92 ± 0.05) × 10^7^ particles per mL (Fig. 3B) at the same camera collection parameters. When increasing the laser power, the size distribution shifts toward smaller sizes (Fig. 3D and E), which implies that the increase in particle concentration observed at higher laser power is due to the emergence of a population of smaller particles. However, there is a simultaneous shift of the residence time distribution to lower times (Fig. 3G and H); the increase in laser power lowers the average residence time from (175 ± 1) ms to (108.7 ± 0.2) ms. A lower average residence time is expected as smaller particles diffuse in and out of the field of view more quickly. However, residence times for the particles detected at the lower power should remain in the residence time distribution with the same high residence times, which is not the case in Fig. 3H. Even particles detected at low power have a reduced residence time.

This conclusion is supported by Fig. 3C, F, and I, where data have been collected at 12 mW laser power using a lower camera exposure time such that the scattering signal is comparable to that of the video taken at low power. This accounts for the possibility that the reduction of residence time is due to the observation of smaller particles at higher laser power. The concentration detected in this video is (2.46 ± 0.03) × 10^7^ particles per mL, a lower concentration similar to that detected at 6 mW due to the decrease in scattering signal collected. Therefore, if the size and residence time distributions depend on laser power because of the scattering signal, then Fig. 3A, D, and G should be similar to Fig. 3C, F, and I, respectively. This is not the case as both the size and residence time distributions retain their shifted shape and resemble that of Fig. 3E and H. This indicates that the size and residence time depressions observed are not caused by the emergence of smaller particles at higher laser power, and some phenomenon causes FFA scatterers to move into and out of the field of view more quickly with increased laser power.

Additionally, the residence time decrease even at a lower detected particle concentration (Fig. 3I) makes it unlikely that tracks are truncated by particle overlap when laser power is increased.^19^ If particles were too close to each other at high laser power and long exposure time, then the residence time would increase at the shorter exposure time, which is not observed. Furthermore, the average nearest neighbour distance of the particles at high laser power and long exposure time is (15 ± 8) μm whilst the search radius during linking between frames is 1.7 μm, making particle overlap rare.

A change in the size distribution is problematic for the interpretation of NTA data at higher laser power, because whilst a higher laser power might reveal smaller particles due to the enhanced detection limit, a shift of the histogram at a higher laser power despite collecting the same scattering signal makes it hard to conclude that higher laser power reveals smaller particles.

## 4. Particles of known size

**Fig. 4:**
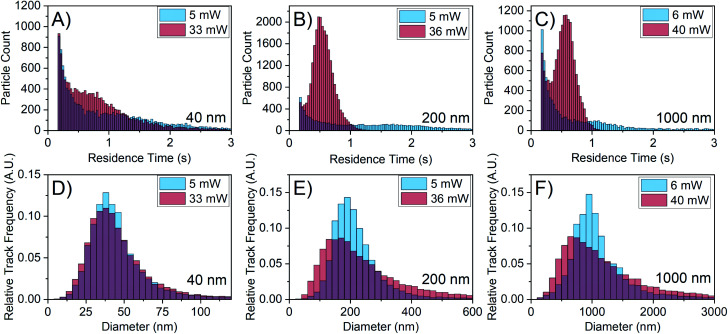
NTA of particles of known size at low and high laser power. The plots are labelled with their corresponding size. (A–C) Residence time distributions showing a shift to the left for all particle sizes. (D–F) Size distributions showing a shift to the left for 200 nm and 1000 nm particles. The minute change in the 40 nm histogram corresponds with the smaller change in the residence time histogram compared to that of the larger particle sizes. The apparent size is reduced when laser power is increased, even for particles of known size.

To eliminate the possibility of the emergence of smaller particles at higher laser power, particles of well-defined size were characterised at different laser powers. Fig. 4 shows the results of NTA with 40 nm gold particles, 200 nm polystyrene particles, and 1000 nm silica particles. At higher laser power, the residence time distribution narrows and shifts to smaller times for all particle sizes as shown in Fig. 4A–C, similar to the observed behaviour of FFA in Fig. 3G and H. More importantly, the size distribution shifts to the left for the 200 nm and 1000 nm particles as shown in Fig. 4E and F. There is also a slight change in the 40 nm particle distribution (Fig. 4D), which matches the comparatively small shift in its residence time distribution. Because the particles in this case are of known size, the increased laser power should not reveal smaller particles but simply increase the amount of scattering signal from the particles. In these experiments, the camera collection parameters have been adjusted to ensure the scattering centres are not overexposed and the video quality remains consistent.

The shift in the histogram observed here for known particle sizes when changing laser power is clearly concerning for the use of NTA in interrogating particles of unknown size *e.g.* FFA mesoscopic species; if a shift in the size distribution is observed for particles of defined size, it becomes very difficult to assess to what degree the histogram shift is caused by the change in the detection limit when interrogating particles of unknown size. Understanding the relationship between reduced residence time and the shift in the size distribution will help remedy this additional shift.

## 5. Sampling error

**Fig. 5:**
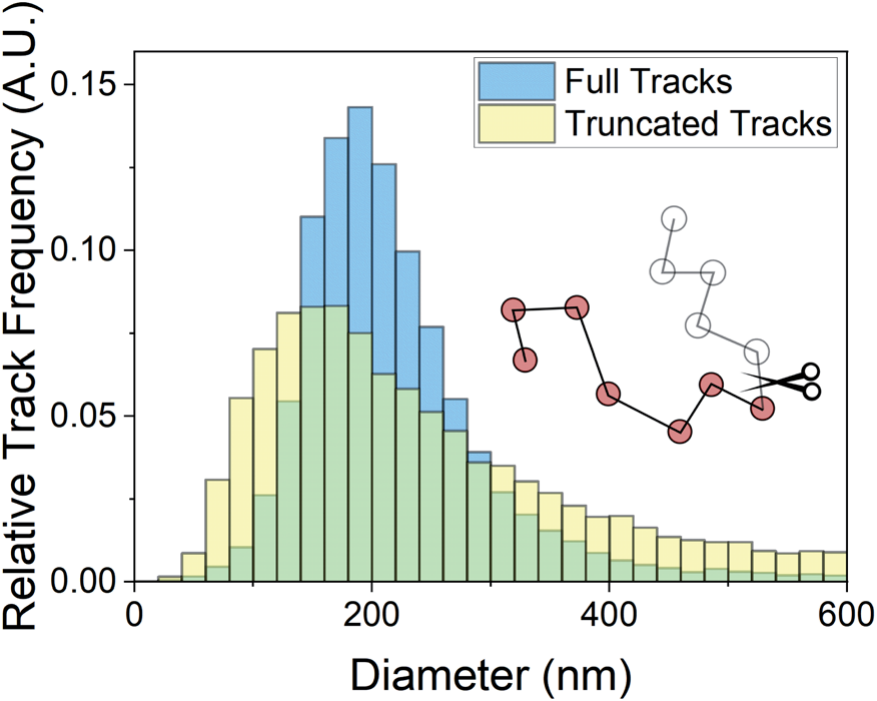
Size distributions of 200 nm particles output by NTA at 5 mW with no restriction on the number of frames a particle is tracked (blue) and a maximum of 17 frames per particle tracked (yellow), matching the average residence time recorded at 36 mW. The histogram shifts left with the track length restriction due to increased sampling error on individual particle mean squared displacements, demonstrating the deleterious effect of reduced residence time on the interpreted size.

Minimum residence time, also known as minimum track length, has been noted previously as an important parameter in NTA analysis. The use of maximum likelihood estimation, which accounts for particle track length in the generation of size distributions, has become a regularly used analysis method.^3,17,20,21^ The dependence of the size distribution on track length stems from sampling error on individual particle displacements.^22^ The particle mean squared displacement used in eqn (2) is related to particle displacements as follows:4
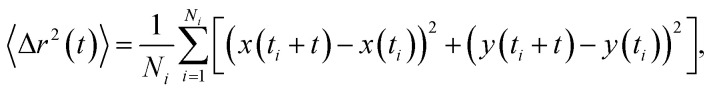
where *t* is the lag time, *t*_*i*_ is the time corresponding to the *i*th image on the particle track, and *N*_*i*_ is the total number of images over which the particle is tracked. The measured values in this equation are *x* and *y*, both of which are normally distributed about zero displacement for any given lag time. The mean squared displacement loses precision for small samplings of displacement, and the error propagates into the linear regression of mean squared displacement *vs.* lag time, from which the diffusion coefficient is derived. Increased error on the diffusion coefficient (and therefore size in eqn (3)) results in a broadening of the size distributions at decreased residence time. This sampling error is modelled in Fig. 5, where size distributions of 200 nm particles characterised at 5 mW have been derived from NTA with (i) no restriction on particle track length and (ii) a 17 frame particle track length maximum imposed. The track length maximum selected matches the average residence time at 36 mW in Fig. 4E. We observe a similar shift to smaller sizes that is present in Fig. 4E, supporting the assertion that this shift is caused by sampling error. Therefore, comparing size distributions at different laser powers requires that the sampling error on the data is comparable so that increased sampling error at high laser power does not mask changes to the distribution due to the improved detection limit. The source of residence time depression must be eliminated so that comparable sampling errors are obtained at different laser powers.

## 6. Laser-induced convection

**Fig. 6:**
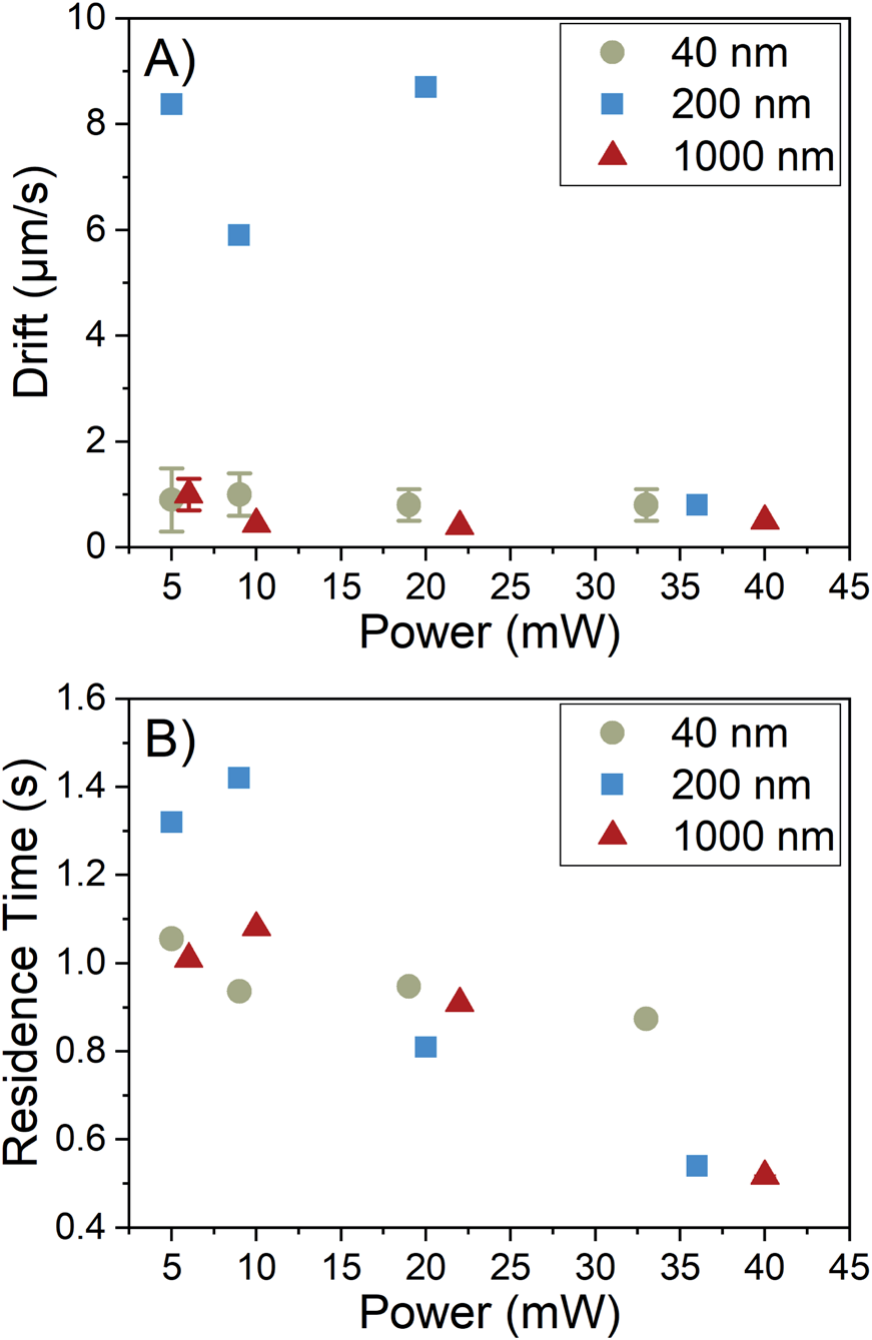
(A) Drift and (B) residence time *vs.* laser power for particles of known size. Whilst there is no trend between drift and laser power, residence time decreases with increased laser power for all particles. This implies that increasing laser power does not enhance in-plane particle motion and only enhances *z*-axis particle motion to reduce residence time. This sole increase in *z*-axis motion is caused by convection, which acts along the axis of gravity.

Residence time is primarily controlled by the particle motion along the axis perpendicular to the image plane, *i.e.* the *z*-axis as shown in Fig. 1A. Particles generally appear in the field of view and then disappear before moving laterally out of the field of view. This indicates that the phenomenon causing the residence time reduction observed acts along the *z*-axis. This conclusion is further supported by Fig. 6A, which shows the average values of drift with increasing laser power. Drift is the bulk in-plane movement of all particles in the field of view which is not due to Brownian motion. We reserve the term “drift” only for motion in the plane of the video, which can be corrected for with the tracking algorithm, and distinguish this from motion out of the video plane, which cannot be corrected for in the same way. In the tracking analysis, drift is subtracted from particle displacements to obtain movement only caused by Brownian motion. There is no correlation between laser power and drift, indicating that the increase in laser power has no noticeable effect on in-plane motion.

However, Fig. 6B shows a downward trend in residence time with an increase in laser power, indicating an enhancement of motion along the *z*-axis. This direction corresponds to the axis along which gravity acts. We conclude that this enhanced motion is due to the development of convection currents acting out of the image plane. Convection currents are caused by density gradients in the fluid, which in this system are likely caused by heating of the solvent by the laser, despite the minimal absorption of light by ethanol at this wavelength. This reasoning is further supported by the residence time depression occurring for all sample types, implying a solvent-mediated motion where particles are carried with the solvent during convection. This effect has also been observed in an optical forces study,^23^ where convection was observed with a detection arm oriented along the *y*-axis, where diffusion along the negative *z*-axis was visible. However, to the best of our knowledge, the effects of convection on residence time resulting in distribution broadening have never been discussed in NTA, with considerations of convection limited to effects on particle diffusion *i.e.* drift.^4,24^ A prediction of motion along this axis using only Brownian motion can be made and compared to residence times observed. Even for 40 nm particles, which are expected to diffuse the quickest, the expected time for a particle to diffuse out from the center of the light sheet with 1/*e*^2^ thickness of 30 μm is approximately 12 s, which is significantly longer than the 1 s average residence time we observe, implying the presence of convection even at low laser power. The presence of convection accounts for the observed reduction of residence time for all particles and the resulting increase in the error of the size distributions.

## 7. Shutter

The laser is simultaneously required to image particles in NTA and the source of the increased error at higher laser power. A solution would involve being able to image the particles whilst avoiding convection by reducing the total amount of energy deposited into the sample. Here we do this by using a shutter to irradiate particles for time periods short enough that convection is reduced. To modify the device for this purpose, a shutter was incorporated in the illumination arm which blocks the beam and unblocks during time periods of video collection, as depicted in Fig. 1A. In the FFA samples, convection increases with the length of time the sample is irradiated on a seconds time scale, so the shutter was set to open and close on this time scale. This method is similar to a possible pulsed laser solution, but it should be noted that in either case, the total energy deposited into the sample should be minimised to reduce convection. An Arduino Uno electronics board was programmed to sequentially open and close the shutter with a 5 s unblocked duration and 15 s blocked duration as a proof of concept. The camera collection time was matched with the shutter *via* a synchronisation link from the Arduino board.

**Fig. 7:**
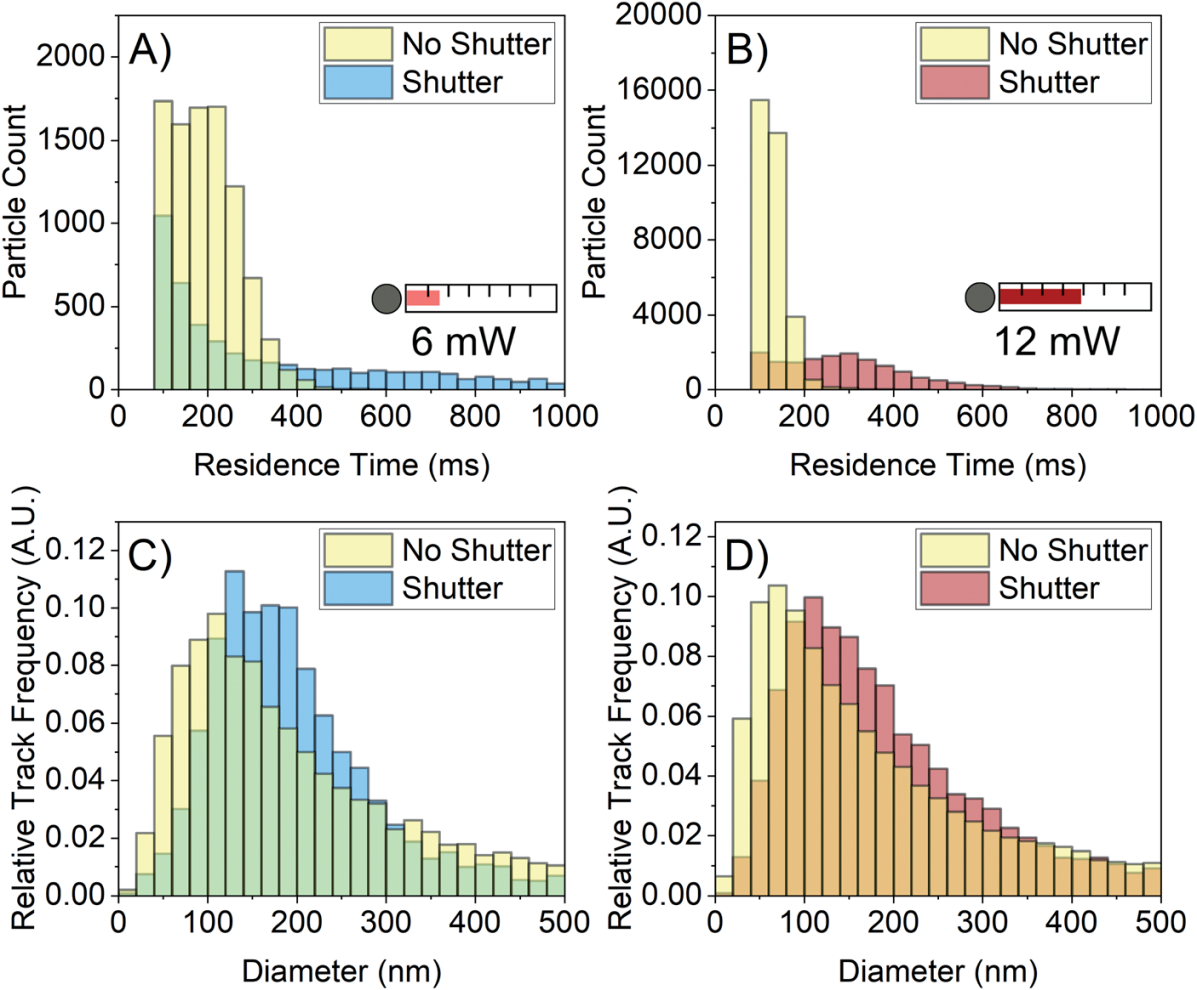
Results of using a shutter to reduce laser-induced convection by lowering the amount of energy deposited into the sample. (A and B) Residence time distributions at 6 mW and 12 mW with the shutter open continuously for the entire video collection (yellow) and the shutter operating with 5 s open, 15 s closed sequence (blue and red). There is a right shift of the residence time distribution out to larger times for both laser powers. (C and D) Corresponding size distributions showing the shift of the size distribution to the right when the shutter sequence is used, increasing the accuracy of the size distribution.

Fig. 7 shows the NTA data collected from 1 M FFA with and without the shutter. For both the 6 mW and 12 mW powers, the residence time distribution shifts right to longer times when the shutter repeatedly blocks and unblocks the laser beam as depicted in Fig. 7A and B. The shutter is decreasing the laser-induced convection, allowing for particles to remain in the field of view for longer residence times. When the shutter is used, the average residence time increases from (175.1 ± 0.8) ms to (407 ± 6) ms for the 6 mW power and from (108.7 ± 0.2) ms to (270 ± 1) ms for the 12 mW power.

There is also a rightward shift of the size distributions for both powers as shown in Fig. 7C and D. With the shutter employed, the modal size bin increases from 100–120 nm to 120–140 nm for the 6 mW power, and from 60–80 nm to 100–120 nm for the 12 mW power. The difference in average residence time between the 6 mW power and the 12 mW power manifests in the shape of their respective size distributions; the 12 mW size distribution retains its leaning shape, implying that the error on this histogram is still relatively large. However, the 6 mW size distribution shows improvement in both shift and shape, implying that the error on this distribution is lower than that of the 12 mW distribution. This matches expectation that higher residence time decreases sampling error and increases the precision of individual particle sizes.

It is important to note that the broadness of the distributions in this work is larger than is typical of NTA data. The present work has shown that this poor precision of the distributions is attributed to sampling error and residence time. Furthermore, in the generation of the FFA histograms, the same criterion on the number of required tracks, 2, was set. The histograms generated from videos where the shutter was used could be made more accurate by increasing the number of required tracks. This improvement, however, is less applicable to videos where the shutter is not used and the residence times of most of the particles are very low. Ongoing is work to refine the shutter blocking and unblocking times to obtain more accurate size distributions.

The incorporation of the shutter has improved the error on the histograms, resulting in the rightward shift of the distributions. Understanding the origin of the histogram broadening as well as finding a solution allows for a more robust comparison of the size histograms at low and high power. The residence time distribution serves as a quality check on the video data collected, and the shutter a mechanism by which the video data can be improved.

## 8. Conclusions

In summary, we have explored the use of a laser with controllable power in NTA and the substantial effect of increasing laser power on the error on the size distribution retrieved with this technique. FFA is used as a case study, because similar systems have recently been studied with NTA. High laser power is shown to enable the detection of more particles as expected, but with the additional effect of reducing the residence time. Particles of known size were interrogated to show that not only is the residence time depression present across particle sizes, but that the error on the size distribution increases. This result has immediate implications in the use of NTA where laser powers in the range of tens of mW are used, like in commercial devices.^25^ The inability to decouple this increase of the error from the improvement of the detection limit due to increased laser power makes it difficult to interpret NTA data at higher laser power.

We show that the origin of this error is track length reduction at high laser power due to residence time depression. It is determined that the cause of residence time depression at higher laser power is convection of the sample, resulting in particles moving through the field of view more quickly. A shutter is presented as a solution, which permits the characterisation of particle diffusion before convection substantially increases the individual particle sampling error. Recent work has also shown that reduction of convection can also be accomplished by restricting the size of the sample volume,^26^ another possible route for increasing laser power and signal without the adverse effect of convection. Our study paves the way for the robust use of NTA on samples where higher laser power grants access to particle populations below the detection limit at low laser power.

## Data access statement

Data collected for this work can be made available upon reasonable request.

## Conflicts of interest

There are no conflicts to declare.

## Supplementary Material
